# The CCN2 Polymorphism rs12526196 Is a Risk Factor for Ascending Thoracic Aortic Aneurysm

**DOI:** 10.3390/ijms232315406

**Published:** 2022-12-06

**Authors:** Antonio Tejera-Muñoz, Isabel Rodríguez, Álvaro Del Río-García, Yamina Mohamedi, María Martín, Valentina Chiminazzo, Beatriz Suárez-Álvarez, Carlos López-Larrea, Marta Ruiz-Ortega, Raúl R. Rodrigues-Díez

**Affiliations:** 1 Molecular and Cellular Biology in Renal and Vascular Pathology, IIS-Fundación Jiménez Díaz, Universidad Autónoma de Madrid, Av Reyes Católicos 2, 28040 Madrid, Spain; 2 Research Support Unit, Hospital General Mancha Centro, 13600 Alcázar de San Juan, Spain; 3 Cardiac Pathology Research Group, Instituto de Investigación Sanitaria del Principado de Asturias (ISPA), 33011 Oviedo, Spain; 4 Cardiology Department, Hospital Universitario Central de Asturias, 33011 Oviedo, Spain; 5 Biostatistics and Epidemiology Platform, Instituto de Investigación Sanitaria del Principado de Asturias (ISPA), 33011 Oviedo, Spain; 6 Translational Immunology, Instituto de Investigación Sanitaria del Principado de Asturias ISPA, 33011 Oviedo, Spain; 7 Servicio de Inmunología, Hospital Universitario Central de Asturias, 33011 Oviedo, Spain

**Keywords:** aneurysm, CCN2, polymorphism, vascular diseases, ascending aorta

## Abstract

Cellular communication network factor 2 (CCN2/CTGF) has been traditionally described as a downstream mediator of other profibrotic factors including transforming growth factor (TGF)-β and angiotensin II. However, recent evidence from our group demonstrated the direct role of CCN2 in maintaining aortic wall homeostasis and acute and lethal aortic aneurysm development induced by angiotensin II in the absence of CCN2 in mice. In order to translate these findings to humans, we evaluated the potential association between three polymorphisms in the *CCN2* gene and the presence of a thoracic aortic aneurysm (TAA). Patients with and without TAA retrospectively selected were genotyped for rs6918698, rs9402373 and rs12526196 polymorphisms related to the *CCN2* gene. Multivariable logistic regression models were performed. In our population of 366 patients (69 with TAA), no associations were found between rs6918698 and rs9402373 and TAA. However, the presence of one C allele from rs12526196 was associated with TAA comparing with the TT genotype, independently of risk factors such as sex, age, hypertension, type of valvulopathy and the presence of a bicuspid aortic valve (OR = 3.17; 95% CI = 1.30–7.88; *p* = 0.011). In conclusion, we demonstrated an association between the C allele of rs12526196 in the CCN2 gene and the presence of TAA. This study extrapolates to humans the relevance of CCN2 in aortic aneurysm observed in mice and postulates, for the first time, a potential protective role to CCN2 in aortic aneurysm pathology. Our results encourage future research to explore new variants in the CCN2 gene that could be predisposed to TAA development.

## 1. Introduction

The cellular communication network factor (CCN) family consists of matricellular proteins sharing a structural tetramodular organization with an essential role regulating extracellular matrix (ECM) homeostasis, as well as other cellular functions [1]. The CCN family is composed of six members (CCN1–6), with CCN2, previously known as connective tissue growth factor (CTGF) [2], being one of the most studied components [1]. Initially, CCN2 was described as a downstream mediator of several profibrotic factors, including transforming growth factor β (TGF-β), angiotensin II and endothelin-1 [3,4,5]. However, over the years, several studies have contributed to modifying this concept by describing CCN2 as a growth factor that directly regulates intracellular pathways implicated in proinflammatory and pro-oxidant responses [6]. In the cardiovascular system, CCN2 has been described as being highly expressed during embryogenesis in branchial arches, the heart and vascular cells of major blood vessels, decreasing its expression in adult tissues [7,8]. However, increased levels of CCN2 have been described in experimental and human cardiovascular diseases, including heart failure, pulmonary hypertension, restenosis, atherosclerosis and aortic aneurysms [7,9], suggesting CCN2 as a potential therapeutic target. Interestingly, we have recently demonstrated that the deletion of *Ccn2* in adult mice alters vascular integrity and functions predisposed to aneurysm formation, which suggests a potential protective role of CCN2 in maintaining the aortic wall homeostasis under pathological conditions [10].

There is a wide range of factors implicated in aneurysm pathophysiology, with abnormal expression or function of key proteins being the leading causes of aneurysm development. In this sense, mutations in genes related to the TGF-β pathway activation, one of the main sources of CCN2 expression, are related to pathologies coursing with aortic aneurysm formation such as Marfan, Loeys–Dietz and Ehlers–Danlos syndromes [11]. Apart from gene mutations, protein levels can also be influenced by single nucleotide polymorphisms (SNPs) in the encoding genes [12]. Previous studies have described the association between SNPs in genes encoding cytokines (IL-6 and TNF-α) and several extracellular matrix related-proteins (matrix metalloproteinases, tissue inhibitors of metalloproteinases, TGF-β, elastin and type III procollagen) and aortic aneurysm susceptibility [13,14]. However, although the association between CCN2 SNPs and other pathologies such as hepatic fibrosis [15], colon carcinoma [16], diabetic nephropathy [17], pulmonary fibrosis [18], systemic sclerosis [19] and in-stent restenosis [20] has been established, the potential association between CCN2 SNPs and aortic aneurysm development still remains unexplored.

Thus, considering our recent and novel results in mice demonstrating the protective role of CCN2 in aneurysm development after systemic angiotensin II infusion, and aiming to further decrypt the role of CCN2 in human aneurysms, in this study we wanted to evaluate the potential association between functional SNPs in the gene encoding CCN2 and susceptibility to aortic aneurysm in patients.

For this purpose, we performed a retrospective transversal cohort study to evaluate the prevalence of the different alleles in three SNPs—rs6918698, rs9402373 and rs12526196, which have been postulated to alter gene transcription or transcript stability [15,19]—and the possible association with the presence of ascending thoracic aortic aneurysm (TAA).

## 2. Results

### 2.1. Genotype and Allele Frequencies from rs6918698, rs9402373 and rs12526196 Polymorphisms Do Not Differ in Thoracic Aortic Aneurism Patients

Out of the 366 recruited patients, 69 patients presented TAA, and 297 did not; the latter were used as controls. Characteristics of both groups are summarized in Table 1.

There were more men in the TAA group, which was younger, with more current smokers; hypertension and diabetes were less frequent, and more aortic valve regurgitation and bicuspid aortic valve (BAV) were present. The whole population was at the Hardy–Weinberg equilibrium for the three polymorphisms. The genotype and allele frequencies of rs6918698, rs9402373 and rs12526196 did not differ in the patient and control groups (Table 2).

### 2.2. rs12526196 SNP Is Associated with Thoracic Aortic Aneurism Independently of Risk Factors

**Figure 1:**
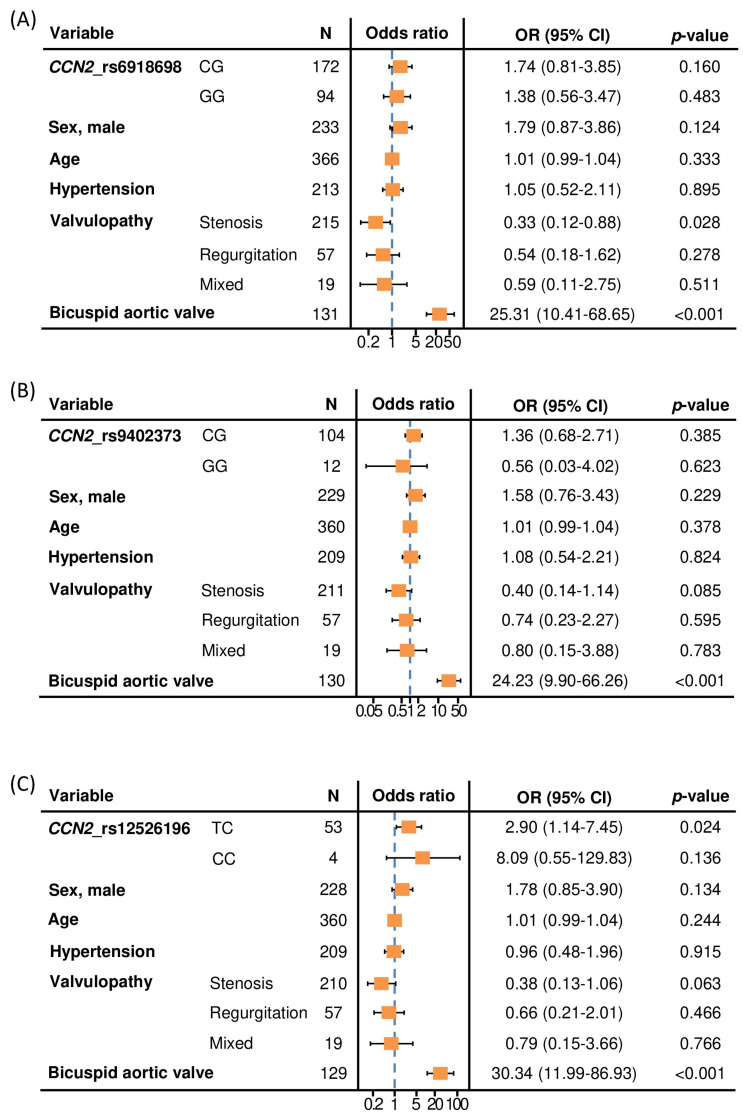
Forest plots showing the multivariable logistic regression models for the association between CCN2 rs6918698 (**A**), rs9402373 (**B**) and rs12526196 (**C**) polymorphisms and the presence of thoracic aortic aneurysm, adjusted for potential confounders. Reference for CCN2_rs6918698 polymorphism: CC genotype. Reference for CCN2_rs9402373 polymorphism: CC genotype. Reference for CCN2_rs12526196 polymorphism: TT genotype. Reference for sex: female. Reference for valvulopathy: normal functioning valve. Reference for bicuspid aortic valve: tricuspid aortic valve. N = number of patients. OR (95% CI): Odds ratio (95% confidence interval).

The multivariable analysis, including several risk factors for the presence of TAA, showed that rs6918698 and rs9402373 polymorphisms were not significantly associated with TAA (Figure 1A,B). However, the TC genotype from the rs12526196 polymorphism was associated with the presence of TAA, compared to patients with the TT genotype (OR = 2.90; 95% CI = 1.14–7.45; *p* = 0.024). This statistically significant association was independent of sex, age, hypertension and type of valvulopathy, and even independent of the presence of BAV, which was the main contributor to the TAA risk (Figure 1C).

**Figure 2:**
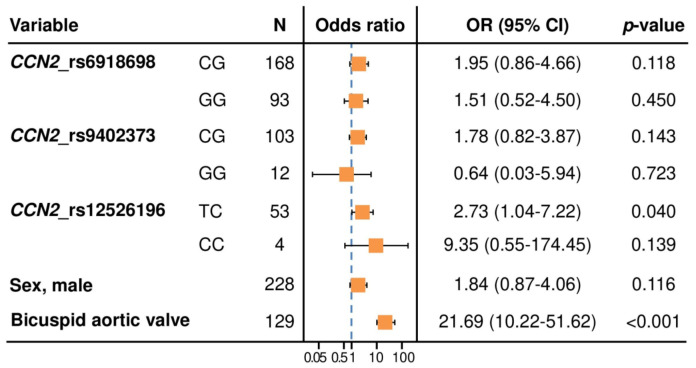
Forest plot showing the multivariable logistic regression model for the association between CCN2 rs12526196 polymorphism and the presence of thoracic aortic aneurysm, adjusted for rs6918698 and rs9402373 polymorphisms and other potential confounders selected with the Akaike information criterion. Reference for CCN2_rs6918698 polymorphism: CC genotype. Reference for CCN2_rs9402373 polymorphism: CC genotype. Reference for CCN2_rs12526196 polymorphism: TT genotype. Reference for sex: female. Reference for bicuspid aortic valve: tricuspid aortic valve. N = number of patients. OR (95% CI): Odds ratio (95% confidence interval).

The association between the TC genotype and the presence of TAA was found even when controlling for the other two polymorphisms (OR = 2.93; 95% CI = 1.10–7.86; *p* = 0.031) (Figure 2).

**Figure 3:**
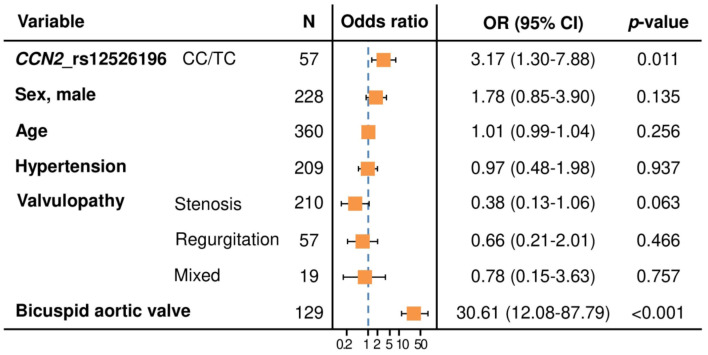
Forest plot showing the multivariable logistic regression model for the association between CCN2 rs12526196 polymorphism, under a dominant model of inheritance, and the presence of thoracic aortic aneurysm, adjusted for potential confounders. Reference for CCN2_rs12526196 polymorphism: TT genotype. Reference for sex: female. Reference for valvulopathy: normal functioning valve. Reference for bicuspid aortic valve: tricuspid aortic valve. N = number of patients. OR (95% CI): Odds ratio (95% confidence interval).

Considering the low prevalence of the homozygous CC genotype from rs12526196 in our cohort and the same effect direction (OR > 1) in both CC and CT genotypes, we performed an additional multivariable analysis according to a dominant model of inheritance, including TC and CC patients in one group. Adjusting by the same confounding variables, this new analysis revealed that the presence of at least one C allele (found in 20.9% of TAA patients, Table 2) was a risk factor for TAA, compared with patients carrying the TT genotype of the CCN2 rs12526196 polymorphism (OR = 3.17; 95% CI = 1.30–7.88; *p* = 0.011) (Figure 3).

## 3. Discussion

Our study demonstrates, for the first time, an association between the presence of the C allele in the rs12526196 SNP in CCN2 and the occurrence of TAA in patients. On the one hand, this result provides a new genetic variant potentially involved in the susceptibility to aortic aneurysms apart from those already known related to other HCTD. Thus, this variant could be taken into account as a tool in the assessment of patients at risk of TAA, such as patients with BAV. On the other hand, these results reinforce the concept of CCN2 as an essential protein maintaining the aortic wall homeostasis, and encourages future studies to search for the relation of other SNPs or mutations in the gene encoding CCN2 that may help to design new tools to predict and/or manage aortic aneurysms.

The relevance of CCN2 regulating the fibrotic response has already been clearly demonstrated in several preclinical studies where the blockage of this protein has shown promising results in improving fibrosis in liver, lung and renal pathologies. Furthermore, there are clinical trials in phase 2 or 3 using anti-CCN2 therapies in Duchenne muscular dystrophy (NCT02606136), pancreatic adenocarcinoma (NCT04229004) and idiopathic pulmonary fibrosis (NCT03955146). However, the role of CCN2 in the cardiovascular system has recently been questioned by several works. Thus, despite the increased levels of CCN2 observed in experimental and human cardiovascular diseases, including heart failure, pulmonary hypertension, restenosis, atherosclerosis or aortic aneurysms [9], the preclinical attempts to regulate CCN2 levels have led into mixed results. In this sense, while CCN2 inhibition using a monoclonal antibody attenuates left ventricular remodeling and dysfunction in pressure overload-induced heart failure [21], CCN2 knockout does not affect cardiac hypertrophy and fibrosis formation upon chronic pressure overload [22], and cardiac-restricted overexpression of CCN2 attenuates left ventricular remodeling after myocardial infarction [23]. Concordantly, the post-ischemic administration of recombinant human CCN2 increased the tolerance of ex vivo-perfused murine hearts to ischemia reperfusion injury [24]. Focusing on blood vessels, our group has recently demonstrated that CCN2 plays an essential role as a protective factor in the angiotensin-induced aortic aneurysm model. In addition, other authors have highlighted the relevance of CCN2 in the regulation of vascular stiffness [25].

CCN2 protein levels can be influenced by SNPs in the encoding gene. Some SNPs in CCN2 have been associated with an increased risk of developing several human pathologies, mainly related to fibrotic disorders, including systemic sclerosis [19], pulmonary fibrosis [18], colon carcinoma [16], diabetic nephropathy [17] and hepatic fibrosis [15]. In the latter, authors analyzed 15.3 kb of upstream sequence and 14.1 kb of downstream sequence of CCN2 gene, where 61 SNPs were found. Among them, rs9402373 was associated with severe hepatic fibrosis in four patient cohorts evaluated, rs12526196 in two, and rs6918698 in one of them [15]. In the subsequent analyses, authors demonstrated, by both in silico and in vitro studies, that rs9402373 and rs12526196 polymorphisms affect nuclear factor binding and may alter CCN2 gene transcription or transcript stability. In this sense, in silico studies predicted that the presence of the T allele in the rs12526196 SNP may specifically bind FOXQ1, FOXL1, SRY and several SOX factors (SOX9, SOX5 and SOX17), while C allele may not specifically bind one transcription factor [15]. Supporting these data, in vitro studies have demonstrated that the rs12526196T allele has a greater binding affinity for nuclear factors than C allele [15]. Remarkably, it has been also described that SOX9 directly induces CCN2 transcription in chondrocytes and nucleus pulposus cells [26], and FOXL1 deletion decreases CCN2 expression in lung fibroblast [27]. Coming back with fibrotic disorders, increased levels of SOX9 have been observed in lung and kidney fibrosis, postulating SOX9 inhibition as a therapeutic target [28,29]. By contrast, reduced SOX9 levels associated with fibulin-5 downregulation have been found in human aortic aneurysms [30], highlighting the different role of this factor depending on the pathology. Altogether, these results suggest that the presence of the C allele in the rs12526196 SNP could be predisposed to TAA development by, at least in part, reducing nuclear factors binding and, therefore, downregulating gene transcription.

This study presents several limitations. First, the limited number of cases analyzed in our study, derived from its retrospective nature that also did not allow us to have all the desirable variables, constitutes one of the main limitations. Second, since we have included only patients with European ancestry, variants could be population-specific and, therefore, validation of these results in other populations is needed in order to confirm this association, and to define this variant as a valuable clinical marker. Third, we cannot rule out confounding from unmeasured variables or other major pathologies that could be associated with this variant, although other severe diseases are unlikely to affect a significant number of patients. Last, the effect of CCN2 SNPs in the circulating levels of the protein has not been evaluated. In this sense, as it has been recently described in an elegant and well conducted study, CCN2 is synthesized and secreted as an inactive preprotein and requires proteolytic processing to become a factor able to elicit cell signaling responses [31]. These results further strengthen the dual role of CCN2 acting not only as a matricellular protein, but also as a growth factor with autocrine and paracrine actions [31]. Therefore, an exhaustive analysis would be necessary to clearly define whether the effect of SNPs on CCN2 expression levels are correlated with increased levels of circulating CCN2 and whether these, in turn, correlated with changes in CCN2 activity.

## 4. Conclusions

Our study enhances the idea of the dual role of CCN2 depending on the pathology. Firstly, we had described that CCN2 deletion led to aortic homeostasis disruption predisposed to aneurysm development. Now, we have supported this predisposition by the finding of an association between the C allele of the rs12526196 functional SNP and the presence of TAA in humans. Although further research into the precise mechanisms regulating CCN2 expression by rs12526196C allele and its role in aortic aneurysm development and progression is needed, these results strength the main role of CCN2 as an essential factor in the aortic wall. Altogether, our study encourages future research to explore new variants in the CCN2 gene that could be predisposed to TAA development.

## 5. Materials and Methods

### 5.1. Study Population

The present retrospective and transversal study included unrelated individuals with self-reported European ancestry referred for study and follow-up or after surgery for aortic valve replacement, ascending aneurysm repair, or both, in a specific consultation of aortic pathology at Hospital Universitario Central de Asturias (HUCA). They were recruited between May 2010 and April 2014. The study was conducted in accordance with the Declaration of Helsinki and the human sample collection protocol was approved by the Ethics Committee for Investigation of the Principality of Asturias (84/13). All patients signed an informed consent form to participate in the study. The selection included patients diagnosed with TAA defined as a permanent localized dilatation of the artery having at least a 50% increase in diameter compared with the expected normal diameter following the current guidelines at that time [32,33]. Aortic diameter was measured by echocardiography and, if any doubt, by computed tomography. As controls, patients referred for an echocardiographic study due to ischemic heart disease, with no previous diagnosis of aortic disease, no family history and no disease predisposed to aortic aneurysm, who showed no evidence of aortic dilatation on the echocardiography, were included. In both cases and controls, diagnoses of aortic valve regurgitation or stenosis and normal functioning aortic valve were recorded [34,35]. Aortic valve morphology was defined by two experimented observers through an echocardiography as having two or three separate functional leaflets or after valve replacement, once valve morphology was confirmed by the surgeon. Excluding criteria comprised patients with suboptimal echocardiographic images and with any doubt in the number of aortic valve leaflets. In addition, patients with previous or a suspicious diagnosis of genetic syndromes such as Marfan syndrome [36], Ehlers–Danlos syndrome [37] or other hereditary connective tissue disorders (HCTD) associated with aortic disease were excluded as well to avoid confusion with the association with other phenotypic features of the syndromes.

Hypertension was defined as the fulfillment of any of the following criteria: clinical history of hypertension; systolic blood pressure ≥ 140 mmHg or diastolic blood pressure ≥ 90 mmHg, in at least two determinations; or antihypertensive treatment that was not administered as therapy to pathology other than arterial hypertension [38]. Hyperlipidemia was defined according to the fulfillment of one of the following criteria: clinical history of hyperlipidemia, total cholesterol levels >200 mg/dL, LDL cholesterol ≥ 130 mg/dL, HDL cholesterol < 40 mg/dL or lipid lowering therapy [39]. The existence of diabetes mellitus was considered based on the presence of any of the following premises: history of diabetes mellitus accredited in a medical report, fasting blood glucose ≥ 200 mg/dL in any situation, symptoms of diabetes mellitus, at least two fasting blood glucose determinations ≥ 126 mg/dL or current use of oral hypoglycemic treatments and/or insulin [40].

### 5.2. Genotyping

Genomic DNA was purified from peripheral blood samples obtained in EDTA tubes, following standard procedures, and was stored at −20 °C. Samples were genotyped by quantitative PCR using Taqman probes (ThermoFisher Scientific, Waltham, MA, USA) for the following CCN2 polymorphisms: rs6918698 (C_27443107_10), rs9402373 (C_30110249_10), and rs12526196 (C_1764938_10). Genotypes were assigned with StepOne Software v2.3. Around 5% of the samples were randomly selected and re-genotyped in order to confirm the accuracy of the genotyping procedure, and no discrepancies were found.

### 5.3. Statistical Analyses

Descriptive statistics was reported as median and interquartile range for continuous variables and absolute numbers and percentages for categorical variables. The Mann–Whitney test and Chi-squared or Fisher’s exact test were performed to compare the distribution of continuous and categorical variables, respectively. Chi-squared test was also used for the assessment of the Hardy–Weinberg equilibrium. The association between each polymorphism and TAA was evaluated using univariable and multivariable logistic regression models to adjust for TAA risk factors (sex, age, hypertension, type of valvulopathy and aortic valve morphology). A multivariable logistic regression model was also performed to analyze by controlling for the other two polymorphisms. Possible confounders among traditional TAA risk factors were identified with a backward selection based on the Akaike information criterion (AIC) in order to avoid overfitting. Results were reported as odds ratio (OR), 95% confidence interval (CI) and *p*-value. *p*-values <0.05 were considered statistically significant. The analyses were performed using R software (version 4.1.3) [41].

## Figures and Tables

**Table 1:** Characteristics of the study population.

Variable	Controls N = 297	TAA N = 69	*p*-Value
**Sex**			0.001
Male	177 (59.6)	56 (81.2)	
Female	120 (40.4)	13 (18.8)	
**Age, years**	69.0 [60.0, 77.0]	58.0 [44.0, 67.0]	<0.001
**Smoking status**			0.035
Never	196 (66.0)	39 (56.5)	
Current	69 (23.2)	26 (37.7)	
Former	32 (10.8)	4 (5.8)	
**Dyslipidemia**	140 (47.1)	26 (37.7)	0.198
**Hypertension**	183 (61.6)	30 (43.5)	0.009
**Diabetes mellitus**	89 (30.0)	3 (4.3)	<0.001
**Aortic valve**			0.002
Normal functioning	64 (21.5)	11 (15.9)	
Stenosis	183 (61.6)	32 (46.4)	
Regurgitation	36 (12.1)	21 (30.4)	
Mixed	14 (4.71)	5 (7.25)	
**Bicuspid aortic valve**	72 (24.2)	59 (85.5)	<0.001

Categoric variables are frequency (%). Quantitative variable is shown as median [interquartile range]. TAA: thoracic aortic aneurysm. Mixed: simultaneous presence of both stenosis and regurgitation. Statistically significant values are shown in bold.

**Table 2:** Univariable logistic regression models for genotype and allele frequencies of the polymorphisms in the study population, according to the presence of thoracic aortic aneurysm.

Polymorphism	Total (nN = 366) ^†^	Controls (N = 297)	TAA (N = 69)	OR (95% CI)	*p*-Value
**CTGF_rs6918698**					
CC	100 (27.3)	85 (28.6)	15 (21.7)	-	
CG	172 (47.0)	135 (45.5)	37 (53.6)	1.55 (0.82–3.08)	0.190
GG	94 (25.7)	77 (25.9)	17 (24.6)	1.25 (0.58–2.70)	0.563
C allele	372 (50.8)	305 (51.3)	67 (48.6)	-	
G allele	360 (49.2)	289 (48.7)	71 (51.4)	1.12 (0.77–1.62)	0.554
**CTGF_rs9402373**					
CC	244 (66.7)	202 (68.9)	42 (62.7)	-	
CG	104 (28.4)	80 (27.3)	24 (35.8)	1.44 (0.81–2.52)	0.203
GG	12 (3.3)	11 (3.8)	1 (1.5)	0.437 (0.02–2.34)	0.434
C allele	592 (82.2)	484 (82.6)	108 (80.6)	-	
G allele	128 (17.8)	102 (17.4)	26 (19.4)	1.14 (0.70–1.82)	0.586
**CTGF_rs12526196**					
TT	303 (82.8)	250 (85.3)	53 (79.1)	-	
TC	53 (14.5)	41 (14.0)	12 (17.9)	1.38 (0.66–2.74)	0.372
CC	4 (1.1)	2 (0.7)	2 (3.0)	4.72 (0.56–40.03)	0.125
T allele	659 (91.5)	541 (92.3)	118 (88.1)	-	
C allele	61 (8.5)	45 (7.7)	16 (11.9)	1.63 (0.87–2.93)	0.113

Variables are shown as frequency (%). ^†^ n = 360 for CTGF_rs9402373 and CTGF_rs12526196 due to missing genotyping. TAA: thoracic aortic aneurysm; OR: Odds ratio; CI: Confidence interval; OR (95% CI) and *p*-value for the comparison between Control and TAA groups. -: Reference.

## Data Availability

The data supporting the results of this study are available from the corresponding authors upon reasonable request.

## References

[1] Leask A., Abraham D.J. (2006). All in the CCN family: Essential matricellular signaling modulators emerge from the bunker. J. Cell Sci..

[2] Perbal B., Tweedie S., Bruford E. (2018). The official unified nomenclature adopted by the HGNC calls for the use of the acronyms, CCN1–6, and discontinuation in the use of CYR61, CTGF, NOV and WISP 1–3 respectively. J. Cell Commun. Signal..

[3] Ruiz-Ortega M., Rodriguez-Vita J., Sanchez-Lopez E., Carvajal G., Egido J. (2007). TGF-β signaling in vascular fibrosis. Cardiovasc. Res..

[4] Rodríguez-Vita J., Sánchez-López E., Esteban V., Rupérez M., Egido J., Ruiz-Ortega M. (2005). Angiotensin II Activates the Smad Pathway in Vascular Smooth Muscle Cells by a Transforming Growth Factor-β–Independent Mechanism. Circulation.

[5] Rupérez M., Lorenzo O., Blanco-Colio L.M., Esteban V., Egido J., Ruiz-Ortega M. (2003). Connective Tissue Growth Factor Is a Mediator of Angiotensin II–Induced Fibrosis. Circulation.

[6] Rodrigues-Diez R.R., Garcia-Redondo A.B., Orejudo M., Rodrigues-Diez R., Briones A.M., Bosch-Panadero E., Kery G., Pato J., Ortiz A., Salaices M. (2015). The C-Terminal Module IV of Connective Tissue Growth Factor, Through EGFR/Nox1 Signaling, Activates the NF-κB Pathway and Proinflammatory Factors in Vascular Smooth Muscle Cells. Antioxid. Redox Signal..

[7] Ponticos M. (2013). Connective tissue growth factor (CCN2) in blood vessels. Vasc. Pharmacol..

[8] Ivkovic S., Yoon B.S., Popoff S.N., Safadi F.F., Libuda D.E., Stephenson R.C., Daluiski A., Lyons K. (2003). Connective tissue growth factor coordinates chondrogenesis and angiogenesis during skeletal development. Development.

[9] Meng Y., Tian C., Liu L., Wang L., Chang Q. (2013). Elevated expression of connective tissue growth factor, osteopontin and increased collagen content in human ascending thoracic aortic aneurysms. Vascular.

[10] Rodrigues-Díez R.R., Tejera-Muñoz A., Esteban V., Steffensen L.B., Rodrigues-Díez R., Orejudo M., Rayego-Mateos S., Falke L.L., Cannata-Ortiz P., Ortiz A. (2022). CCN2 (Cellular Communication Network Factor 2) Deletion Alters Vascular Integrity and Function Predisposing to Aneurysm Formation. Hypertension.

[11] Ostberg N., Zafar M., Ziganshin B., Elefteriades J. (2020). The Genetics of Thoracic Aortic Aneurysms and Dissection: A Clinical Perspective. Biomolecules.

[12] Pinard A., Jones G.T., Milewicz D.M. (2019). Genetics of Thoracic and Abdominal Aortic Diseases. Circ. Res..

[13] Ogata T., Shibamura H., Tromp G., Sinha M., Goddard K.A., Sakalihasan N., Limet R., MacKean G.L., Arthur C., Sueda T. (2005). Genetic analysis of polymorphisms in biologically relevant candidate genes in patients with abdominal aortic aneurysms. J. Vasc. Surg..

[14] Jabłońska A., Zagrapan B., Neumayer C., Eilenberg W., Scheuba A., Brostjan C., Demyanets S., Klinger M., Nanobachvili J., Huk I. (2020). Polymorphisms in the IL-6 and TNF-α gene are associated with an increased risk of abdominal aortic aneurysm. Int. J. Cardiol..

[15] Dessein A., Chevillard C., Arnaud V., Hou X., Hamdoun A.A., Dessein H., He H., Abdelmaboud S.A., Luo X., Li J. (2009). Variants of CTGF are associated with hepatic fibrosis in Chinese, Sudanese, and Brazilians infected with Schistosomes. J. Exp. Med..

[16] Ahmad A., Askari S., Befekadu R., Hahn-Strömberg V. (2014). Investigating the association between polymorphisms in connective tissue growth factor and susceptibility to colon carcinoma. Mol. Med. Rep..

[17] Wang B., Carter R.E., Jaffa M.A., Nakerakanti S., Lackland D., Lopes-Virella M., Trojanowska M., Luttrell L., Jaffa A.A. (2010). The DCCT/EDIC Study Group Genetic variant in the promoter of connective tissue growth factor gene confers susceptibility to nephropathy in type 1 diabetes. J. Med. Genet..

[18] Klay D., van der Vis J.J., Roothaan S.M., Nguyen T.Q., Grutters J.C., Goldschmeding R., van Moorsel C.H.M. (2021). Connective Tissue Growth Factor Single Nucleotide Polymorphisms in (Familial) Pulmonary Fibrosis and Connective Tissue Disease Associated Interstitial Lung Disease. Lung.

[19] Fonseca C., Lindahl G.E., Ponticos M., Sestini P., Renzoni E.A., Holmes A.M., Spagnolo P., Pantelidis P., Leoni P., McHugh N. (2007). A Polymorphism in the *CTGF* Promoter Region Associated with Systemic Sclerosis. N. Engl. J. Med..

[20] Bujak K., Lejawa M., Gąsior M., Osadnik T. (2020). The CTGF gene -945 G/C polymorphism is associated with target lesion revascularization for in-stent restenosis. Exp. Mol. Pathol..

[21] Szabó Z., Magga J., Alakoski T., Ulvila J., Piuhola J., Vainio L., Kivirikko K.I., Vuolteenaho O., Ruskoaho H., Lipson K. (2014). Connective Tissue Growth Factor Inhibition Attenuates Left Ventricular Remodeling and Dysfunction in Pressure Overload–Induced Heart Failure. Hypertension.

[22] Fontes M.S., Kessler E.L., van Stuijvenberg L., Brans M.A., Falke L.L., Kok B., Leask A., van Rijen H.V., Vos M.A., Goldschmeding R. (2015). CTGF knockout does not affect cardiac hypertrophy and fibrosis formation upon chronic pressure overload. J. Mol. Cell. Cardiol..

[23] Gravning J., Ørn S., Kaasbøll O.J., Martinov V.N., Manhenke C., Dickstein K., Edvardsen T., Attramadal H., Ahmed M.S. (2012). Myocardial Connective Tissue Growth Factor (CCN2/CTGF) Attenuates Left Ventricular Remodeling after Myocardial Infarction. PLoS ONE.

[24] Kaasbøll O.J., Moe I.T., Ahmed M.S., Stang E., Hagelin E.M.V., Attramadal H. (2016). CTGF/CCN2 Postconditioning Increases Tolerance of Murine Hearts towards Ischemia-Reperfusion Injury. PLoS ONE.

[25] Chaqour B. (2019). Caught between a “Rho” and a hard place: Are CCN1/CYR61 and CCN2/CTGF the arbiters of microvascular stiffness?. J. Cell Commun. Signal..

[26] Oh C.-D., Yasuda H., Zhao W., Henry S.P., Zhang Z., Xue M., De Crombrugghe B., Chen D. (2016). SOX9 directly Regulates CTGF/CCN2 Transcription in Growth Plate Chondrocytes and in Nucleus Pulposus Cells of Intervertebral Disc. Sci. Rep..

[27] Miyashita N., Horie M., Suzuki H.I., Saito M., Mikami Y., Okuda K., Boucher R.C., Suzukawa M., Hebisawa A., Saito A. (2020). FOXL1 Regulates Lung Fibroblast Function via Multiple Mechanisms. Am. J. Respir. Cell Mol. Biol..

[28] Raza S., Jokl E., Pritchett J., Martin K., Su K., Simpson K., Birchall L., Mullan A.F., Athwal V.S., Doherty D.T. (2021). SOX9 is required for kidney fibrosis and activates NAV3 to drive renal myofibroblast function. Sci. Signal..

[29] Gajjala P.R., Kasam R.K., Soundararajan D., Sinner D., Huang S.K., Jegga A.G., Madala S.K. (2021). Dysregulated overexpression of Sox9 induces fibroblast activation in pulmonary fibrosis. JCI Insight.

[30] Orriols M., Varona S., Martí-Pàmies I., Galán M., Guadall A., Escudero J.R., Martín-Ventura J.L., Camacho M., Vila L., Martínez-González J. (2016). Down-regulation of Fibulin-5 is associated with aortic dilation: Role of inflammation and epigenetics. Cardiovasc. Res..

[31] Kaasbøll O.J., Gadicherla A.K., Wang J.-H., Monsen V.T., Hagelin E.M.V., Dong M.-Q., Attramadal H. (2018). Connective tissue growth factor (CCN2) is a matricellular preproprotein controlled by proteolytic activation. J. Biol. Chem..

[32] Hiratzka L.F., Bakris G.L., Beckman J., Bersin R.M., Carr V.F., Casey D., Eagle K.A., Hermann L.K., Isselbacher E.M., Writing Group Members (2010). 2010 ACCF/AHA/AATS/ACR/ASA/SCA/SCAI/SIR/STS/SVM guidelines for the diagnosis and management of patients with Thoracic Aortic Disease: A report of the American College of Cardiology Foundation/American Heart Association Task Force on Practice Guidelines, American Association for Thoracic Surgery, American College of Radiology, American Stroke Association, Society of Cardiovascular Anesthesiologists, Society for Cardiovascular Angiography and Interventions, Society of Interventional Radiology, Soc. Circulation.

[33] Lang R.M., Badano L.P., Mor-Avi V., Afilalo J., Armstrong A., Ernande L., Flachskampf F.A., Foster E., Goldstein S.A., Kuznetsova T. (2015). Recommendations for Cardiac Chamber Quantification by Echocardiography in Adults: An Update from the American Society of Echocardiography and the European Association of Cardiovascular Imaging. J. Am. Soc. Echocardiogr..

[34] Lancellotti P., Tribouilloy C., Hagendorff A., Moura L., Popescu B.A., Agricola E., Monin J.-L., Pierard L.A., Badano L., Zamorano J.L. (2010). European Association of Echocardiography recommendations for the assessment of valvular regurgitation. Part 1: Aortic and pulmonary regurgitation (native valve disease). Eur. Heart J.-Cardiovasc. Imaging.

[35] Baumgartner H., Hung J., Bermejo J., Chambers J.B., Evangelista A., Griffin B.P., Iung B., Otto C.M., Pellikka P.A., Quiñones M. (2009). Echocardiographic Assessment of Valve Stenosis: EAE/ASE Recommendations for Clinical Practice. J. Am. Soc. Echocardiogr..

[36] Loeys B.L., Dietz H.C., Braverman A.C., Callewaert B.L., De Backer J., Devereux R.B., Hilhorst-Hofstee Y., Jondeau G., Faivre L., Milewicz D.M. (2010). The revised Ghent nosology for the Marfan syndrome. J. Med. Genet..

[37] Ehlers-Danlos Syndromes: Revised Nosology, Villefranche, 1997. Ehlers-Danlos National Foundation (USA) and Ehlers-Danlos Support Group (UK)-PubMed. https://pubmed.ncbi.nlm.nih.gov/9557891/.

[38] Mancia G., Fagard R., Narkiewicz K., Redon J., Zanchetti A., Böhm M., Christiaens T., Cífková R., De Backer G., Dominiczak A. (2013). 2013 ESH/ESC Practice Guidelines for the Management of Arterial Hypertension. Blood Press..

[39] Stone N.J., Robinson J.G., Lichtenstein A.H., Merz C.N.B., Blum C.B., Eckel R.H., Goldberg A.C., Gordon D., Levy D., Lloyd-Jones D.M. (2014). 2013 ACC/AHA guideline on the treatment of blood cholesterol to reduce atherosclerotic cardiovascular risk in adults: A report of the American College of Cardiology/American Heart Association Task Force on Practice Guidelines. J. Am. Coll. Cardiol..

[40] American Diabetes Association (2010). Diagnosis and Classification of Diabetes Mellitus. Diabetes Care.

[41] R Core Team (2012). R: A Language and Environment for Statistical Computing.

